# Renal arterial resistive index versus novel biomarkers for the early prediction of sepsis-associated acute kidney injury

**DOI:** 10.1007/s11739-024-03558-y

**Published:** 2024-03-06

**Authors:** Taysser Zaitoun, Mohamed Megahed, Hesham Elghoneimy, Doaa M. Emara, Ibrahim Elsayed, Islam Ahmed

**Affiliations:** 1 Critical Care Medicine Department, Faculty of Medicine, Alexandria University, Alexandria, Egypt; 2 Internal Medicine Department, Faculty of Medicine, Alexandria University, Alexandria, Egypt; 3 Radiodiagnosis and Interventional Radiology Department, Faculty of Medicine, Alexandria University, Alexandria, Egypt; 4 Critical Care Medicine Department, Faculty of Medicine, KFS University, Kafrelsheikh, Egypt; 5 Public Health and Community Medicine Department, Faculty of Medicine, Suez-Canal University, Ismaili, Egypt; 6 Pharmacy Practice and Clinical Pharmacy Department, Faculty of Pharmacy, King Salman International University, South-Sinai, Egypt

**Keywords:** Sepsis, Acute kidney injury, Sepsis-associated acute kidney injury, NGAL, Renal resistive index, Cystatin C, Diagnosis, Sensitivity and specificity

## Abstract

Acute kidney injury (AKI) is a critical complication of sepsis. There is a continuous need to identify and validate biomarkers for early detection. Serum and urinary biomarkers have been investigated, such as neutrophil gelatinase associated lipocalin (NGAL) and cystatin C (Cys C), but their reliability in the intensive care unit (ICU) remains unknown. Renal hemodynamics can be investigated by measuring the renal resistive index (RRI). This study aimed to compare the performance of RRI, serum NGAL (sNGAL), urinary NGAL (uNGAL), and serum Cys C levels as early predictors of the diagnosis and persistence of sepsis-associated AKI. A total of 166 adult patients with sepsis syndrome were enrolled immediately after ICU admission. Biomarkers were measured directly (T1) and on day 3 (T3). RRI was measured directly (T1) and 24 h later (T2). Patients were categorized (according to the occurrence and persistence of AKI within the first 7 days) into three groups: no AKI, transient AKI, and persistent AKI. The incidence rate of sepsis-associated AKI was 60.2%. Sixty-six patients were categorized as in the no AKI group, while another 61 were in transient AKI and only 39 were in persistent AKI. The RRI value (T1 ≥ 0.72) was the best tool for predicting AKI diagnosis (area under the receiver operating characteristic curve, AUROC = 0.905). Cys C (T1 ≥ 15.1 mg/l) was the best tool to predict the persistence of AKI (AUROC = 0.977). RRI (T1) was the best predictive tool for sepsis-associated AKI, while Cys C was the best predictor of its persistence and 28-day mortality.

## Background

Acute kidney injury (AKI) is a common critical complication of sepsis with an incidence ranging from 33 to 50%. As well as sepsis, sepsis-associated AKI is independently associated with increased mortality and morbidity [1]. AKI is a complex and variable clinical illness of several subtypes [2]. The precise mechanism causing sepsis-related AKI is still being researched. It has been suggested that aberrant cellular responses to damage, immunological and autonomic dysregulation, and macrovascular and microvascular dysfunction exist. AKI brought on by sepsis is accompanied by normal or even enhanced renal blood flow [3].

The traditional diagnosis of AKI is based on oliguria and elevated serum creatinine (Scr), which have a number of significant drawbacks [4]. Despite difficulties with diagnosis, it is still not possible to early determine if an AKI is temporary or persistent [5]. Due to a number of factors, the AKI’s duration is quite crucial. The progression to chronic kidney disease (CKD), end stage renal disease (ESRD), and mortality are correlated to the persistency and length of AKI. Early detection and management of those at risk for persistent AKI can be made possible [6, 7].

An important role in the pathophysiological pathways of sepsis-associated AKI may be played by vascular dysfunction. Renal hemodynamics can be investigated without invasive procedures by measuring the renal resistive index (RRI). In the literature, RRI has been used to evaluate the renal perfusion, forecast kidney dysfunction, and monitor AKI recovery [8].

For the purpose of identifying persistent AKI, various urobiological indicators have been investigated but their efficiency in critically ill patients is uncertain. Thus, new biomarkers for the early identification of persistent AKI should be identified and validated. Neutrophil gelatinase-associated lipocalin (NGAL), a 25-kDa protein, was originally isolated from neutrophils and has been extensively investigated in studies to predict the diagnosis of AKI and its subtypes [9]. Cystatin C (Cys C), a 13-kDa cysteine proteinase inhibitor produced by nucleated cells, is filtered by the glomerulus, reabsorbed but not secreted by the renal tubules. Cys C has been found to be better than Scr in detecting small GFR reductions as a renal functional biomarker [9].

The aim of this study is to compare the performance of RRI, serum NGAL (sNGAL), urinary NGAL (uNGAL), and serum Cys C as early predictors of the diagnosis and persistence of sepsis-associated AKI in critically ill patients.

## Methods

After approval from the Medical Ethics Committee of the Faculty of Medicine of Alexandria University (IRB number: 00012098, FWA number: 00018699), protocol registration on clinicaltrials.gov (NCT03799159, protocol ID: RRIBIOSAKI), and informed written consent from the patient’s next of kin (legal guardian), the enrollment process was initiated. This study was conducted according to the Standard for Reporting Diagnostic Accuracy (STARD).

**Fig. 1:**
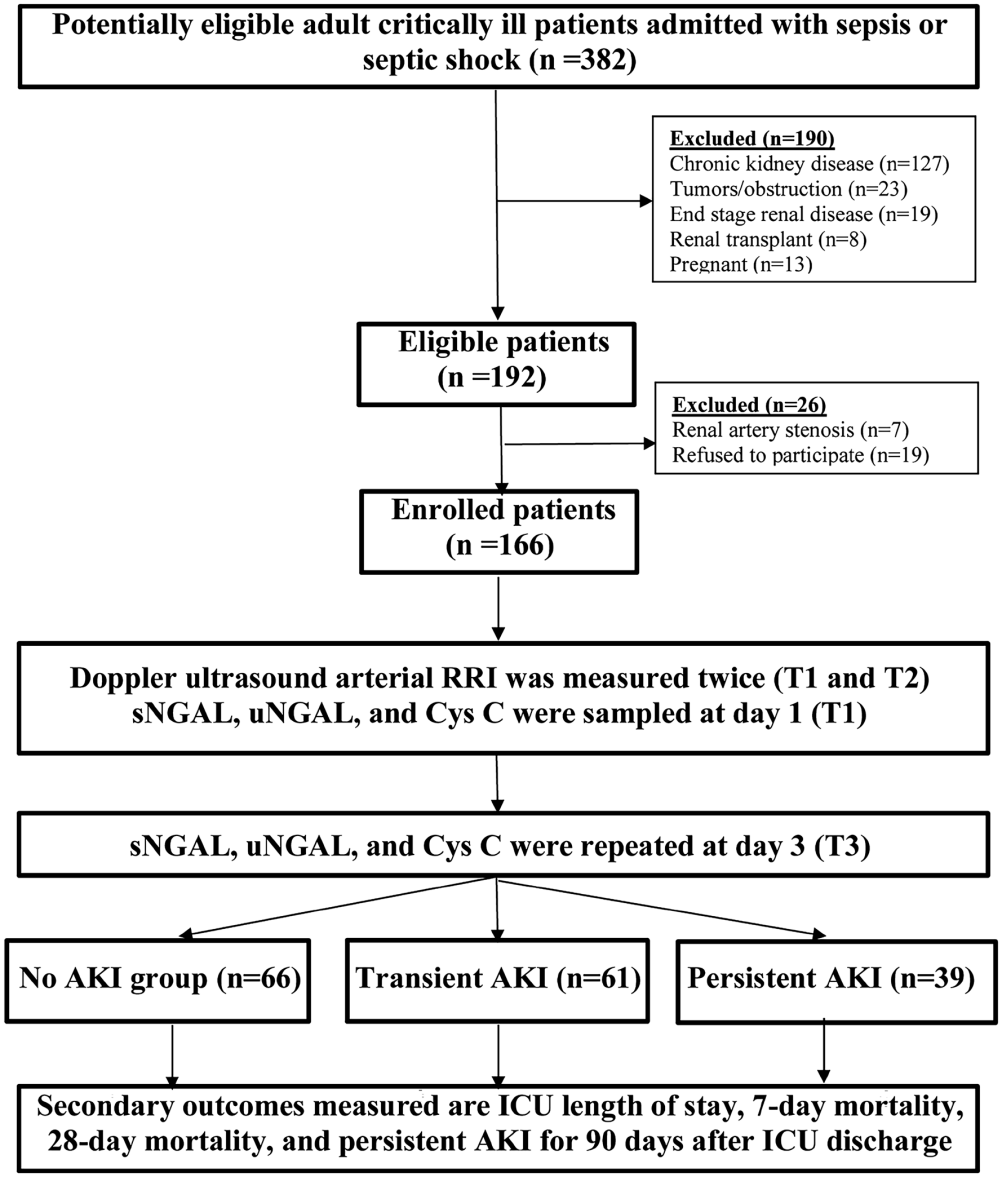
The follow chart of RRIBIOSAKI study

All patients who were eligible for the study were adults (≥ 18 years) and were directly assessed for enrollment after ICU admission with sepsis syndromes (within 2 h) at the critical care units of Alexandria University from May 15, 2019, to February 28, 2021. Pregnancy, renal transplant, renal tumor, nephrectomy, CKD, ESRD, renal artery stenosis, and obstructive uropathy were the exclusion criteria (Fig. 1).

In this single-center prospective cohort, consecutive patients were assessed directly using the following: history taking, physical examination, Glasgow coma scale (GCS), sequential organ failure assessment (SOFA) score, acute physiology and chronic health evaluation (APACHE II) score, routine laboratory investigations, coagulation profile, C-reactive protein (CRP), procalcitonin, and complete sepsis workup to identify the possible sources of sepsis.

Serum and urinary samples were collected directly at the time of enrollment within 2 h of ICU admission (T1) and on day 3 (T3) to measure NGAL (Bioassay Technology Laboratory, E1719Hu) and Serum Cys C (Bioassay Technology Laboratory, E1104Hu).

Ultrasound examinations of the kidneys were performed, including gray-scale ultrasound (size, echogenicity, CKD, or hydronephrosis) and renal Doppler (CX50 CompactXtreme Ultrasound System, Philips, MA, USA, 2–5 MHz curved array transducer)**.** RRI was measured at the time of enrollment (T1) and 24 h later (T2). The RRI of the right kidney was calculated for most of the cohorts. All measurements were performed by the same examiner and repeated by an independent blinded radiologist. Using pulse-wave Doppler, an interlobar or arcuate artery was selected. RRI was calculated as the equation “(PSV-EDV)/PSV”. The means of three distinct RRI calculations were recorded.

All patients received standard in-hospital treatment for sepsis. The treatment protocol was maintained throughout the study. Renal function was monitored and recorded on days 1 (T1), four (T4), and seven (T7). The primary outcome was the occurrence and persistence of AKI within the first 7 days. The patients were categorized according to their primary outcome into three groups: no AKI, transient AKI, and persistent AKI. AKI was classified according to the “Kidney Disease Improving Global Outcomes” (KDIGO). Transient AKI was defined as “AKI with a cause of renal hypoperfusion and recovery within 3 days after inclusion”. Recovery from AKI was defined as “urine output normalization and/or Scr decrease by 50% and/or Scr normalization to its measured or estimated baseline level”. Persistent AKI was defined as a persistent rise in Scr or oliguria after 3 days”. The secondary outcomes were 7-day mortality, 28-day mortality (both measured as all-cause mortality rate), ICU length of stay, and persistence of AKI for 90 days after ICU discharge.

### Statistical analysis

Data was fed to the computer and analyzed using the IBM SPSS (IBM Corp., Armonk, NY, USA) package version 26.0. The significance of the obtained results was determined at the 5% level. The statistical tests used were Kolmogorov Smirnov, Levene’s test, chi-square, Monte Carlo correction, Kruskal Wallis Test, One-way Analysis of variance, Bonferroni correction, and Hosmer Lemeshow goodness of fit model. The Receiver operating characteristic (ROC) curve was generated by plotting sensitivity on the Y axis versus 1-specificity on the x-axis at different cutoff values. The area under the ROC curve (AUROC) denotes the diagnostic performance of the test.

## Results

In this study, 382 eligible patients were screened. After applying the exclusion criteria, only 192 patients were assessed for enrollment. Seven more patients were excluded due to renal artery stenosis and 19 were excluded due to a lack of informed consent. A total of 166 patients were enrolled and included in the final analysis. The median age of the overall cohort was 54 years, and both sexes were equally represented; 50.6% were males. After 7 days of follow-up, the patients were categorized into three groups: no AKI (n = 66), transient AKI (n = 61), and persistent AKI (n = 39) (Table 1).

**Table 1:** Baseline characteristics of the three studied groups

	No AKI (n = 66)	Transient AKI (n = 61)	Persistent AKI (n = 39)	p value
Sex (male)	36 (54.5)	27 (44.3)	21 (53.8)	0.459
Age (years)	57 (10)	51 (10)	53 (14)	0.065
Medical history				
HTN	23 (34.8)	27 (69.2)	22 (56.4)	0.097
DM	16 (24.2)	30 (76.9)	10 (25.6)	0.006*
IHD	13 (19.7)	14 (22.9)	8 (20.5)	0.900
Stroke	13 (19.7)	11 (18.0)	9 (23.1)	0.826
Hepatic	16 (24.2)	7 (11.5)	8 (20.5)	0.172
Chronic AF	12 (18.2)	5 (8.2)	6 (15.4)	0.253
Recent surgery	7 (10.6)	7 (11.8)	5 (12.8)	0.943
COPD	6 (9.1)	4 (6.6)	4 (10.3)	0.786
Malignancy	3 (4.5)	–	–	0.099
Source of sepsis				
Pneumonia	29 (43.9)	27 (44.3)	14 (35.9)	0.663
Mixed	10 (15.2)	4 (6.6)	7 (17.9)	0.181
UTI	7 (10.6)	11 (18.0)	3 (7.7)	0.257
SBP	8 (12.1)	3 (4.9)	5 (12.8)	0.289
CRBSI	4 (6.1)	4 (6.6)	4 (10.3)	0.702
Cellulitis	2 (3.0)	4 (6.6)	3 (7.7)	0.527
Abdomen	2 (3.0)	4 (6.6)	3 (7.7)	0.559
DFI	3 (4.5)	5 (8.2)	–	0.174
GCS	13 (3)	14 (4)	11 (4)	0.054
SOFA score	5 (3)	6 (4)	9 (3)	< 0.001*
Sig. bet. groups	p1 = 0.001*, p2 < 0.0001*, p3 = 0.001*			
APACHE II score	20 (6)	25 (10)	26 (11)	< 0.001*
Sig. bet. groups	p1 < 0.0001*, p2 < 0.0001*, *p3 = 0.53*			
Sepsis	49 (74.2)	33 (54.1)	15 (38.5)	< 0.0001*
Septic shock	17 (25.8)	28 (45.9)	24 (61.5)	
AKI stage 1	–	21 (34.4)	8 (20.5)	< 0.0001*
AKI stage 2	–	5 (8.2)	16 (41.0)	
AKI stage 3	–	35 (57.4)	15 (38.5)	
Vasopressor use	13 (19.7)	19 (31.1)	21 (53.8)	0.001*

Categorical variables are expressed as number (percentage) and compared using the Chi-square test

Non-normally distributed variables are expressed as median (interquartile range) and compared using Kruskal–Wallis’s test

*HTN* hypertension, *DM* diabetes mellitus, *IHD* ischemic heart disease, *AF* atrial fibrillation, *COPD* chronic obstructive pulmonary disease, *UTI* urinary tract infections, *SBP* spontaneous bacterial peritonitis, *CRBSI* catheter-related blood stream infections, *DFI* diabetic foot infections, *GCS* Glasgow coma scale, *SOFA* sequential organ failure assessment score, *APACHE II* acute physiology and chronic health evaluation version II score

p1: p value for comparing between no AKI group and transient group

p2: p value for comparing between no AKI group and persistent group

p3: p value for comparing between transient group and persistent group

*Statistically significant at p ≤ 0.05

The urine output (UOP) was measured during the study period. There were statistically significant differences among the three groups (*p* < 0.001) on days 1, 4, and 7. 100% of patients in the no AKI group had a UOP of more than 0.5 ml/kg/h from day 1 until day 7. 50.8% of patients in the transient AKI group had a UOP of less than 0.5 ml/kg/h on day 1, and all patients in the same group had a UOP of more than 0.5 ml/kg/h at days 4 and 7. 44.5% of patients in the persistent AKI group had a UOP of less than 0.5 ml/kg/h at day 1. 61.5 Of the patients in the persistent AKI group, 61.5% had anuria on day 4, and on day 7, this percentage was elevated to 74.5%. All the measured investigations are shown in Table 2.

**Table 2:** The measured investigations in the three studied groups

	No AKI (n = 66)	Transient AKI (n = 61)	Persistent AKI (n = 39)	p value
Hb (g/dl)	10.5 ± 2.5	10.4 ± 2.4	10.5 ± 2.3	0.965
WBCs (× 103 cells/µl)	16.0 (12.5)	17.0 (10.5)	18.0 (18.0)	0.638
PLTs (× 103/µl)	213.5 (164)	228 (165)	229 (110)	0.138
INR	1.35 (0.41)	1.42 (0.34)	1.35 (0.45)	0.272
Lactate (mmol/l)	5.0 (3.3)	5.0 (4.0)	5.0 (4.0)	0.766
PCT (ng/ml)	2.27 (1.98)	2.36 (1.98)	2.19 (1.62)	0.530
CRP (mg/l)	117 ± 50.8	116 ± 55.9	119 ± 52.5	0.976
Sodium (mmol/l)	133.5 (12)	132.5 (12)	134 (14)	0.297
Potassium (mmol/l)	3.9 ± 0.878	4.1 ± 0.853	4.1 ± 0.800	0.387
BUN T1 (mg/dl)	60 (35)	139 (64)	182 (141)	< 0.001*
Sig. bet. groups	p1 < 0.001*, p2 < 0.001*, *p3 = 1.000*			
BUN T4 (mg/dl)	57 (34)	107 (62)	196 (171)	< 0.001*
Sig. bet. groups	p1 < 0.001*, p2 < 0.001*, p3 = 0.01*			
BUN T7 (mg/dl)	54 (38)	78 (45)	162 (213)	< 0.001*
Sig. bet. groups	p1 < 0.001*, p2 < 0.001*, p3 = 0.001*			
Scr T1 (mg/dl)	0.9 (0.3)	4.3 (4.2)	6.3 (4.5)	< 0.001*
Sig. bet. groups	p1 < 0.001*, p2 < 0.001*, *p3 = 0.334*			
Scr T4 (mg/dl)	0.9 (0.3)	2.3 (1.3)	6.0 (6.1)	< 0.001*
Sig. bet. groups	p1 < 0.001*, p2 < 0.001*, p3 < 0.001*			
Scr T7 (mg/dl)	1.0 (0.3)	1.9 (1.3)	8.5 (6.2)	< 0.001*
Sig. bet. groups	p1 < 0.001*, p2 < 0.001*, p3 < 0.001*			
sNGAL T1 (ng/ml)	322 (305)	620 (466)	582 (489)	< 0.001*
Sig. bet. groups	p1 < 0.001*, p2 < 0.001*, *p3 = 1.000*			
sNGAL T3 (ng/ml)	322 (257)	638 (303)	804 (294)	< 0.001*
Sig. bet. groups	p1 < 0.001*, p2 < 0.001*, *p3 = 0.151*			
uNGAL T1 (ng/ml)	264 (241)	479 (405)	503 (411)	0.002*
Sig. bet. groups	p1 = 0.019*, p2 = 0.004*, *p3 = 1.000*			
uNGAL T3 (ng/ml)	267 (285)	802 (213)	725 (323)	< 0.001*
Sig. bet. groups	p1 < 0.001*, p2 < 0.001*, *p3 = 0.789*			
Cys C T1 (µg/ml)	8.8 (2.4)	11.2 (4.3)	20.4 (3.0)	< 0.001*
Sig. bet. groups	p1 = 0.003*, p2 < 0.001*, p3 < 0.001*			
Cys C T3 (µg/ml)	7.6 (2.9)	16.2 (1.2)	20.2 (8.9)	< 0.001*
Sig. bet. groups	p1 < 0.001*, p2 < 0.001*, *p3 = 0.681*			
RRI (T1)	0.70 (0.03)	0.74 (0.05)	0.76 (0.06)	< 0.0001*
Sig. bet. groups	p1 < 0.0001*, p2 < 0.0001*, p3 = 0.021*			
RRI (T2)	0.72 (0.05)	0.76 (0.05)	0.81 (0.09)	< 0.0001*
Sig. bet. groups	p1 < 0.0001*, p2 < 0.0001*, p3 = 0.002*			

Normally distributed variables are expressed as mean ± standard deviation and compared using one-way ANOVA test

Non-normally distributed variables are expressed as median (interquartile range) and compared using Kruskal–Wallis’s test

*Hb* hemoglobin level, *WBCs* white blood cells count, *PLTs* platelets count, *INR* international normalized ratio, *PCT* procalcitonin, *CRP* C-reactive protein, *BUN* blood urea nitrogen, *Scr* serum creatinine, *sNGAL* serum neutrophile gelatinase associated-lipocalin, *uNGAL* urine neutrophile gelatinase associated-lipocalin, *Cys C* serum cystatin C, *RRI* renal resistive index

p1: p value for comparing between the no AKI group and the transient AKI group

p2: p value for comparing between the no AKI group and the persistent AKI group

p3: p value for comparing between the transient AKI group and the persistent AKI group

**Fig. 2:**
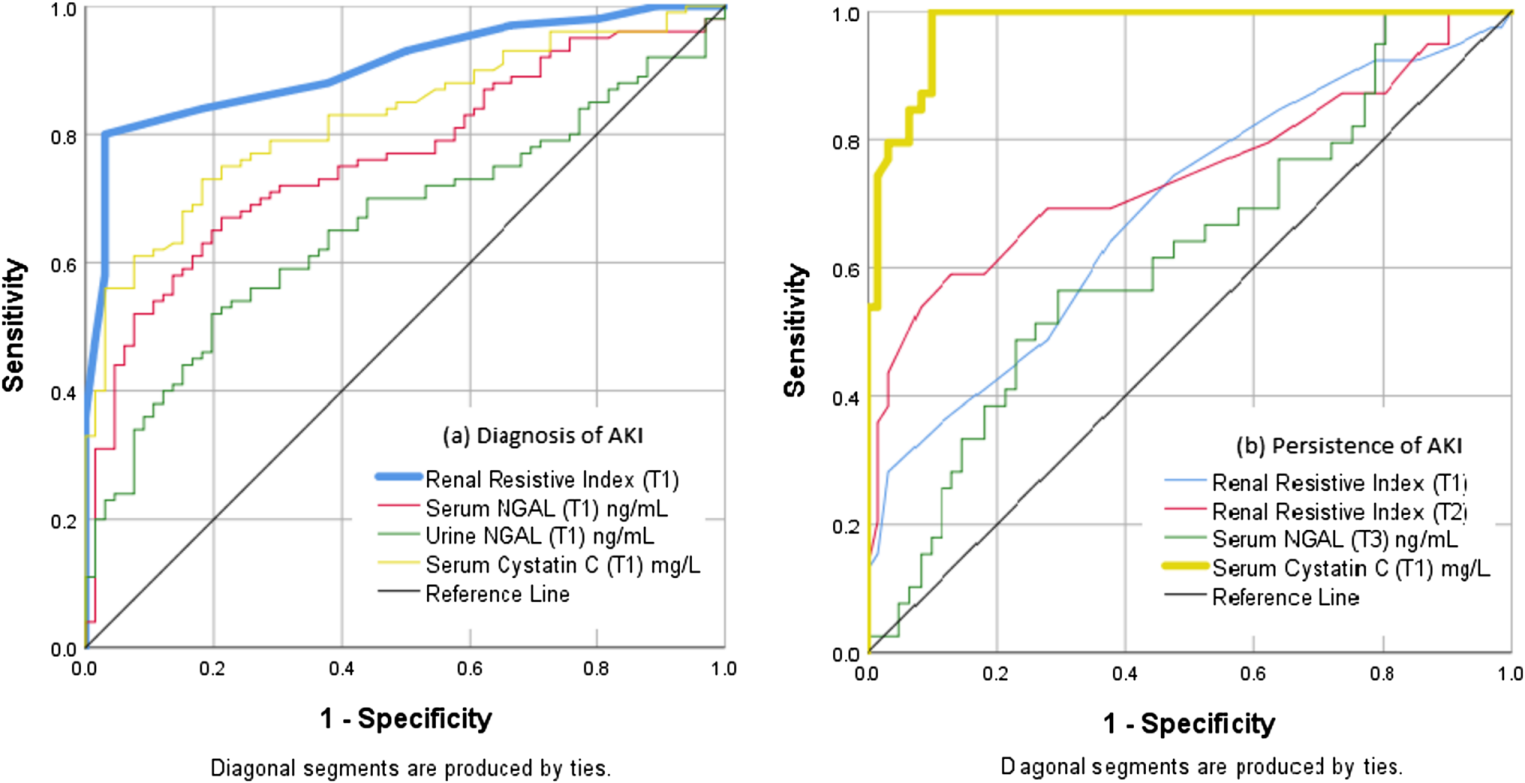
Receiver operating characteristics curve of the measured parameters to predict **a** the diagnosis of AKI and **b** the persistence of AKI

The median RRI upon admission (T1) was significantly higher in the transient AKI group (0.74 ± 0.05, *p*_1_ < 0.0001) and the persistent AKI (0.76 ± 0.06, *p*_2_ < 0.0001) group than the no AKI group (0.70 ± 0.03). The persistent AKI group had a significantly higher median RRI than the transient AKI group (*p*_3_ = 0.021). At day 2 (T2), the median RRI of the transient AKI group (0.76 ± 0.05, *p*_1_ < 0.0001) and the persistent AKI group (0.81 ± 0.09, *p*_2_ < 0.0001) than the no AKI group (0.72 ± 0.05). The persistent AKI group had a significantly higher median RRI than transient AKI group (*p*_3_ = 0.002). In this study, the RRI value (T1) was the best tool to predict AKI at a cutoff value of equal to or greater than 0.72 (AUROC = 0.905, 95% CI 0.860–0.951). RRI showed a positive likelihood ratio of 5.0, which reflects a moderate increase (about 30%) in the probability of having sepsis-associated AKI in patients with RRI ≥ 0.72 upon admission. In terms of differentiating persistent AKI from transient AKI, the RRI value (T1) was a poor tool to predict the persistence of AKI at a cutoff value of equal to or greater than 0.79 (AUROC = 0.687, 95% CI 0.579–0.796). The RRI value (T2) was a good tool to predict the persistence of AKI at a cutoff value of equal to or greater than 0.78 (AUROC = 0.746, 95% CI 0.638–0.853) (Table 3; Fig. 2).

**Table 3:** The predictive performance of the measured study parameters

Diagnosis of AKI	Cutoff value	AUROC	p value	95% CI		+LR	−LR	Sensitivity	Specificity	PPV	NPV	Accuracy
				LL	UL							
RRI (T1)	≥ 0.72	0.905	< 0.001*	0.860	0.951	5.0	0.2	84.2	83.1	88.3	77.6	83.7
sNGAL (T1)	≥ 250	0.763	< 0.001*	0.691	0.835	1.4	0.3	87.0	37.9	67.9	65.8	67.5
uNGAL (T1)	≥ 475	0.659	0.001*	0.577	0.741	2.6	0.6	52.0	80.3	80.0	52.5	63.3
Cys C (T1)	≥ 10.5	0.825	< 0.001*	0.764	0.887	3.5	0.3	75.0	78.8	84.3	67.6	76.5
Persistence of AKI												
RRI (T1)	≥ 0.79	0.687	0.002*	0.579	0.796	3.1	0.7	35.9	88.5	66.7	68.4	68.0
RRI (T2)	≥ 0.78	0.746	< 0.001*	0.638	0.853	2.5	0.4	69.2	72.1	61.4	78.6	71.0
sNGAL (T1)	≥ 250	0.466	0.565	0.351	0.580	1.1	0.7	89.7	14.8	40.2	69.2	44.0
sNGAL (T3)	≥ 780	0.624	0.037*	0.511	0.736	1.9	0.6	56.4	70.5	55.0	71.7	65.0
uNGAL (T1)	≥ 475	0.544	0.460	0.428	0.660	1.1	0.9	53.9	49.2	40.4	62.2	51.1
uNGAL (T3)	≥ 475	0.412	0.139	0.297	0.527	0.9	2.1	79.5	9.8	36.1	42.9	37.0
Cys C (T1)	≥ 15.1	0.977	< 0.001*	0.954	1.000	10	0	100	90.2	86.7	100	94.0
Cys C (T3)	≥ 19.4	0.621	0.052	0.479	0.763	–	0.4	56.1	100	100	78.2	83.0

The Youden index was used to find the optimal cut point to maximize specificity + sensitivity − 1

*AUROC* area under the receiver operating characteristics curve, *p value* probability value, *CI* confidence intervals, *LL* lower limit, *UL* upper limit, *+LR* positive likelihood ratio, *−LR* negative likelihood ratio, *PPV* positive predictive value, *NPV* negative predictive value

*Statistically significant at p ≤ 0.05

The median sNGAL (T1) of the transient AKI (620 ± 466 ng/ml, *p*_*1*_ < 0.001) and persistent AKI (582 ± 489 ng/ml, *p*_2_ < 0.001) groups were significantly higher than the no AKI group (322 ± 305 ng/ml). Both the transient AKI and persistent AKI groups had nearly comparable sNGAL (T1) medians (*p*_3_ = 1.000). Also, the median sNGAL (T3) levels of the transient AKI (638 ± 303 ng/ml, *p*_*1*_ < 0.001) and persistent AKI (804 ± 294 ng/ml, *p*_2_ < 0.001) groups were significantly higher than those of the no AKI group (322 ± 257 ng/ml). The transient AKI had a lower median sNGAL (T3) than the persistent AKI group but was not significant (*p*_3_ = 0.151). Results showed that the sNGAL (T1) at a cutoff value ≥ 250 ng/ml was a good tool, but not the best, to predict sepsis-associated AKI (AUROC = 0.763, 95% CI 0.691–0.835, *p* < 0.001). But the two-time measurements (T1 and T3) of sNGAL have failed to predict the persistence of AKI.

The initial median uNGAL (T1) of the transient AKI (479 ± 405 ng/ml, *p*_*1*_ = *0.019*) and persistent AKI (503 ± 411 ng/ml, *p*_2_ = 0.004) groups were significantly higher than the no AKI group (264 ± 241 ng/ml). Both the transient AKI and persistent AKI groups had nearly comparable uNGAL (T1) medians (*p*_3_ = 1.000). On day 3, the median uNGAL (T3) of the transient AKI (802 ± 213 ng/ml, *p*_*1*_ < 0.001) and persistent AKI (725 ± 323 ng/ml, *p*_2_ < 0.001) groups were significantly higher than the no AKI group (267 ± 285 ng/ml). Both the transient AKI and persistent AKI groups had nearly comparable uNGAL (T3) medians (*p*_3_ = 0.789). Results showed that the uNGAL level at a cutoff value of equal to or more than 475 ng/ml was a poor tool to predict sepsis-associated AKI (AUROC = 0.659, 95% CI 0.577–0.741, *p* = 0.001).

The initial median serum Cys C of the transient AKI (11.2 ± 4.3 µg/ml, *p*_*1*_ = 0.003) and persistent AKI (20.4 ± 3.0 µg/ml, *p*_2_ < 0.001) groups were significantly higher than the no AKI group (8.8 ± 2.4 µg/ml). Also, the persistent AKI group had a significantly higher median Cys C (T1) than the transient AKI group (*p*_3_ < 0.001). Results showed that the Cys C level (T1) at a cutoff value of equal to or more than 10.5 µg/ml was a very good tool to predict sepsis-associated AKI (AUROC = 0.825, 95% CI 0.764–0.887, *p* < 0.001). The Cys C (T1) was the best tool to predict the persistence of AKI at a cutoff value of equal to or greater than 15.1 mg/l (AUROC = 0.977, 95% CI 0.954–1.000) with negative predictive value of 100%. It reflects that the initial Cys C was a good negative marker to exclude the persistence of AKI. On day 3, the median Cys C of the transient AKI (16.2 ± 1.2 µg/ml, *p*_*1*_ < 0.001) and persistent AKI (20.2 ± 8.9 µg/ml, *p*_2_ < 0.001) groups were significantly higher than the no AKI group (7.6 ± 2.9 µg/ml). Both the transient and persistent AKI groups had nearly comparable Cys C (T3) medians (*p*_3_ = 0.681). Also, the Cys C (T3) level failed to predict the persistence of AKI.

Regarding secondary outcomes, the median ICU length of stay for the overall cohort was 7.5 days. This was nearly comparable among the three groups (*p* = 0.162). The 28-day mortality rate of the overall cohort was 34.3%. The persistent AKI group had a significantly higher 28-day mortality rate (58.9%) than the transient AKI (40.9%) and no AKI (13.6%) (*p* < 0.001). Only 18% of the patients in the persistent AKI group had persistent AKI 90 days after enrollment (*p* < 0.001). The need for RRT was significantly higher in the persistent AKI group (51.3%) than in the transient AKI group (19.7%) (*p* < 0.0001). After binary logistic regression, the Cys C levels and APACHE II score were the only predictors of 28-day mortality.

## Discussion

In this study, the early RRI value (T1) was the best tool to predict AKI but a very poor tool to predict its persistence. The second RRI value (T2) was a good tool to predict the persistence of AKI. The diagnostic ability of RRI in the early diagnosis of sepsis-associated AKI in critically ill patients was reported in multiple early studies [10–12]. Another study in 2018 supported its role in the diagnosis of sepsis-associated AKI [13]. In AKI, various factors like inflammation, ischemia, and tubular injury decrease blood flow and increase vascular resistance. This translates to a higher RRI. Therefore, an elevated RRI reflects early changes in renal hemodynamics, even before creatinine rises. In three recent studies, RRI was a poor tool to predict persistent AKI in septic, acute cardiac failure, and general critically ill patients [8, 14, 15].

In contrast to these findings, most of the early studies supported the ability of RRI to predict persistent AKI [16]. A recent prospective observational study showed that the RRI measured within 12 h of admission was a good predictor for persistent sepsis-associated AKI [17]. A meta-analysis (2015) of nine studies showed that an elevated RRI was associated with an increased risk of persistent AKI (OR = 29.85, 95% CI 8.73–102.16; *p* < 0.001) but with significant heterogeneity [5]. In these studies, there was great heterogeneity. Several methodological differences exist, ranging from differences in the definition of transient AKI to differences in methodological qualities. Most of the published studies evaluated the performance of RRI in small sample sizes, making them liable to an overestimation of the size effect. Also, most of the studies evaluated the performance of RRI in the presence of patients without AKI, not between the transient AKI and the persistent AKI groups only. These results may be explained by the poor correlation between RRI and renal vascular resistance as well as the clinically insignificant clinical translation of supraphysiological differences in resistance [18].

RRI results may be impacted by a number of confounding variables, such as vascular compliance, MAP, HR, hypoxemia, and hypercapnia [8]. Vascular compliance was a key factor in determining RRI in initial studies [19]. Reduced vascular compliance can be caused by acute alterations in the intra-abdominal pressure, renal central vasculature, or renal interstitial pressures in addition to underlying subclinical vascular stiffness from age or comorbid conditions, such as HTN, or DM [20–24]. The effect of intra-abdominal pressure on RRI may also result from the possibility that the higher intra-abdominal pressure reduces renal perfusion [25]. Only in cases where vascular compliance was normal did the association between RRI and renal vascular resistance appear to be linear [26]. An earlier report revealed that rather than specific pathophysiological pathways, a longer duration of AKI may indicate a more severe form of the disease [27]. RRI, as a preliminary evaluation of microvascular alterations in the kidney, may not be able to forecast renal recovery due to the complexity of AKI. According to Zhi et al. [14], RRI was not a reliable indicator of stage 3 AKI severity.

In this study, sNGAL (T1) was a good tool, but not the best, to predict sepsis-associated AKI, but the two-time measurements of sNGAL have failed to predict the persistence of AKI. In a pilot study by Lee et al. [28], sNGAL level upon admission (≥ 413.2 ng/ml) was an excellent tool to predict septic-AKI (AUROC = 0.991, *p* < 0.001). Also, in a study by Khawaja et al. [29], the plasma NGAL level at 12 and 24 h after the admission with sepsis were very good tools to predict AKI. These findings were supported by a meta-analysis in 2016 [30], the diagnostic OR of NGAL for predicting sepsis-associated AKI was 6.64 (95% CI 3.80–11.58). Regarding the persistent AKI, similar findings were reported in Zhang et al. [31] study where sNGAL was a poor tool to predict of severe AKI (KDIGO ≥ 2).

In contrast, a recent study by Pei et al. [9] showed that the sNGAL (≥ 95.6 ng/ml) was a poor tool to detect AKI after sepsis (AUROC = 0.620, 95% CI 0.529–0.711). In the same study, serum creatinine and Cys C were better predictors for AKI after sepsis than sNGAL. Also, Kim et al. [32], showed that the plasma NGAL level at a cutoff value of 493 ng/ml was a poor tool (AUROC = 0.675, 95% CI 0.599–0.746) for the prediction of sepsis-associated AKI. The possible explanation for these findings is that NGAL is produced by tissues other than the kidney, such as neutrophils, which synthesize NGAL’s homodimer [30, 33]. Therefore, NGAL may be elevated with inflammation and systemic infections without AKI [31].

Regarding uNGAL in the current study, Results showed that the uNGAL level was a poor tool to predict sepsis-associated AKI or its persistence. In Kim et al. [30] meta-analysis, results showed that the uNGAL has been failed to predict sepsis-associated AKI. These findings may be explained due to poor sensitivity and specificity due to multiple reasons. NGAL levels can be elevated in various conditions besides AKI, including infections, inflammatory states, and CKD. uNGAL level can be affected by other factors like dehydration, and use of diuretics.

In contrast to these findings, three studies showed the uNGAL value at admission was a good valuable tool to predict sepsis-associated AKI [28, 34, 35] and severe AKI (KDIGO ≥ 2) [31]. The explanation for these differences may be reflected by different causes. The calculated fractional NGAL excretion of more than 100% provides additional evidence that differences in NGAL kinetics between serum and urine levels may be caused by local production and excretion of NGAL in the distal tubules [36]. The majority of studies that evaluated the value of uNGAL used various non-standardized assays [37]. In general, oliguric or anuric patients would not benefit from urinary biomarkers. Spot urine samples exhibit great variability and are impacted by various circumstances, such as resuscitation [30]. Also, the presence of different forms of uNGAL, secreted by the renal epithelium and neutrophils [38]. A study showed two peak patterns of uNGAL with two different molecular sizes. Authors suggested that the used polyclonal antibody assay in studies gave different results from the monoclonal antibody assay [39]. In our study, the used ELISA kit was based on a biotinylated NGAL monoclonal antibody.

Regarding CyS C in the current study, the initial Cys C level (T1) was a very good tool to predict sepsis-associated AKI and the best tool to predict its persistence. The Cys C (T3) level failed to predict the persistence of AKI. Our explanation for this point is that the reduction of Cys C levels after hemodialysis was not evaluated, which might affect the accuracy of the Cys C (T3) level between the transient and persistent AKI groups. A prior study showed a marked reduction in the plasma Cys C level during hemodialysis [40]. In accordance with these findings, multiple prior studies supported the same results in both the diagnosis of sepsis-associated AKI or its persistence [9, 41–45]. This good prediction power may be explained by the great characteristics of Cys C, when compared to other markers, such as independent filtration, specificity, and relative stability.

In contrast to these findings, Lee et al. [28] showed that the mean Cys C level (upon admission) was elevated in patients with sepsis-associated AKI (1290.4 ± 222.3 ng/ml) than in the no AKI group (756.3 ± 138.9 ng/ml) with no significant difference (*p* = 0.062). It is important to mention that this study was a pilot study with a small sample size and a high probability of selection bias.

In terms of mortality, the Cys C level was the only predictor of 28-day mortality in our study. RRI, sNGAL, and uNGAL have failed to be independent predictors for 28-day mortality. These findings may raise the importance of using Cys C in Risk stratification models to allow for targeted interventions and potentially improving outcomes.

In contrast to these findings, different findings were previously reported in the literature. Boddi et al. [16] showed that RRI (≥ 0.77) was an independent risk factor for ICU death (OR = 1.63, 95% CI 1.06–2.49, *p* = 0.025) and the strongest predictor for AKI mortality. Wu et al. [46] showed that a high sNGAL level (≥ 250 ng/ml) at ICU admission with sepsis-associated AKI was an independent risk factor for 28-day mortality (OR = 1.072, 95% CI 1.043—1.101, *p* < 0.01). In Khawaja et al. study [29], the median plasma NGAL (12 h) in the non-survivors’ group (170, 95% CI 202–117 ng/ml) was significantly higher than the survivor’s group (123, 95% CI 170–91 ng/ml). Although a small sample size (n = 46) enrolled, a great difference in mortality was detected. In Lee et al. [28] study, sNGAL level (≥ 385.3 ng/ml) was a good tool to predict in-hospital mortality (AUROC = 0.768, *p* = 0.021). The uNGAL level (≥ 383.7 ng/ml) was a good tool to predict in-hospital mortality (AUROC = 0.780, *p* = 0.016). Cys C was comparable between survivors and non-survivors (*p* = 0.076).

To summarize this point, there is no ideal marker to predict sepsis-associated AKI-associated mortality. Most of these studies lack the sample size necessary to identify mortality differences. They are diagnostic studies with small sample sizes. Some of these studies enrolled non septic patients and healthy volunteers in the overall sample. The measured outcomes varied between 28-day mortality, AKI mortality, ICU mortality, and in-hospital mortality.

### Limitations

This study has several limitations. Firstly, the monocentric design may affect external validity. The principal investigator was not blinded to the patient baseline characteristics and the diagnostic study design. The RRI is influenced by some risk factors, such as DM, atherosclerosis, and HTN [47, 48]. RRI was not measured prior to resuscitation. The value of RRI might be unstable due to the impact of changes in MAP prior to and after resuscitation. The effect of positive end-expiratory pressure and peak airway pressure on RRI was not considered, which would lead to an elevated RRI measurement. The majority of measured biomarkers take time to obtain and are not used in clinical practice. The possible effect of the central venous pressure (CVP) was not considered due to the partial unavailability of central venous catheters, especially in patients with sepsis. An inferior vena cava evaluation (caliper and collapsibility) can be evaluated and associated to the RI evaluation in another study. Reports showed an association between the existence of new or persistent AKI in sepsis and high CVP measurement. The interaction between the SOFA score and sNGAL levels was not explored. Some studies showed that low SOFA score was associated with a larger mean difference of sNGAL between the sepsis-associated AKI and the no AKI group. The thyroid dysfunction was not investigated, although there is weak but not definitive evidence that thyroid dysfunction in critically ill population may affect the ability of Cys C in detecting the diagnosis of AKI.

## Conclusions

According to the results of this study, in critically ill patients with sepsis syndromes, the Doppler ultrasound arterial RRI upon ICU admission was the best predictive tool for sepsis-associated AKI. The Cystatin C levels upon admission and at day 3, were the best predictors for the persistence of sepsis-associated AKI and 28-day mortality, respectively. Although serum and urine NGAL showed fair diagnostic abilities, both failed to predict the persistence of sepsis-associated AKI or 28-day mortality.

Multicentric studies and investigator-blinded designs are recommended for future research. The prognostic ability of renal biomarkers and RRI to detect the need for RRT should be investigated. More prognostic and risk stratification studies in this point are recommended for further research. All these study limitations should be considered. Combinations between the measured study parameters and the classic diagnostic tools should be evaluated to enhance the diagnostic or prognostic performance of single markers.

## Data Availability

The data that support the findings of this study are available on reasonable request from the corresponding author, [T.Z].
